# Correlation between Epstein–Barr virus and anti-cytomegalovirus/-herpes simplex virus/-*Toxoplasma gondii* antibodies in Chongqing, China: a cross-sectional observational study

**DOI:** 10.3389/fmed.2025.1661969

**Published:** 2025-11-05

**Authors:** Youyu Zhang, Shupeng Song, Yilin Wu, Beining Ding, Xuenuo Chen, Qian Li, Yuxia Du, Huiling Liu, Yongguo Li

**Affiliations:** Department of Infectious Diseases, The First Affiliated Hospital of Chongqing Medical University, Chongqing, China

**Keywords:** Epstein–Barr virus, cytomegalovirus, *Toxoplasma gondii*, positivity rate, EBV reactivation

## Abstract

**Background:**

Epstein–Barr virus (EBV) infects more than 95% of the global population, and EBV reactivation is associated with the development of various diseases. The aims of this study were (1) to investigate the epidemiology of EBV, CMV, HSV, and *Toxoplasma gondii* infections and their associations with serum antibody profiles and (2) to explore the relationships between EBV infection and reactivation and the antibody status of *Toxoplasma gondii*, CMV, and HSV.

**Methods:**

This retrospective study detected EBV-specific antibody profiles or plasma EBV-DNA, as well as antibodies against *Toxoplasma gondii*, cytomegalovirus (CMV), and herpes simplex virus (HSV). Basic demographic information, including age and sex, was collected to assess the EBV infection status and the prevalence of antibodies against *Toxoplasma gondii*, CMV, and HSV in different populations.

**Results:**

A total of 3,046 hospitalized patients (1,524 male, 1,522 female) who underwent antibody testing for *Toxoplasma gondii*, CMV, and HSV were included in the study. The overall serum positivity rates for *Toxoplasma gondii* IgG, CMV IgG, and HSV IgG increased with age, with overall rates of 16.84, 97.50, and 91.20%, respectively. Among the 1,079 patients who underwent EBV-DNA testing, the lowest virus detection rate (9.97%) was found in the 21–40-year-old age group, with a progressively increasing rate with age. Additionally, compared with patients who were negative for *Toxoplasma gondii* IgG, those with *Toxoplasma gondii* IgG positivity had significantly higher rates of EBV-EA-IgG and EBV-VCA-IgA antibody positivity (*p* = 0.032; *p* < 0.001). Furthermore, patients with EBV reactivation had the highest CMV IgM antibody positivity rate (60.53%), followed by those with primary EBV infection (45.45%), whereas patients without EBV infection had the lowest rate (25.75%), with statistically significant differences between the groups.

**Conclusion:**

EBV antibody profiles positivity rates were higher in patients with *Toxoplasma gondii* IgG positivity than in those with *Toxoplasma gondii* IgG negativity. The CMV IgM antibody positivity rate was significantly higher in EBV reactivation group than in other groups. These results highlight potential diagnostic relevance of co-testing for EBV and CMV in suspected reactivation cases.

## 1 Introduction

Epstein–Barr virus (EBV) is an oncogenic *γ*-herpesvirus virus containing 172 kb of double-stranded linear DNA that was originally identified in 1964 in patients with Burkitt’s lymphoma (BL) (1). EBV infects its primary host target, human B cells, by binding to complement 47 receptors 1 and 2 and MHC class II (2). It continues to infect more than 95% of the adult population worldwide (3). Primary EBV infections are usually asymptomatic, occur in childhood, and are then carried in the body for life (4). EBV infection can lead to a variety of diseases. During adolescence or adulthood, 30–50% of infections manifest as infectious mononucleosis (IM) (1). Globally, EBV causes more than 200,000 cancers annually, including Hodgkin’s lymphoma, non-Hodgkin’s lymphoma, central nervous system (CNS) lymphoma in patients with late-stage HIV infection, posttransplant lymphoproliferative disease (PTLD), nasopharyngeal carcinoma, and gastric carcinoma (5, 6). Therefore, further research on EBV is critically important for human health.

Latency and reactivation are hallmarks of herpesvirus infection. In almost all EBV-infected B cells, viral infection occurs in a latent state (7), establishing a lifelong latent infection within the host lymphoid tissue (8). EBV reactivation hinges on activation of lytic genes (BZLF1, BRLF1), triggering viral replication. Clinical confirmation requires elevated EBV DNA load (qPCR) or serological rises in anti-EA (IgG/IgM) and anti-VCA (IgM) antibodies (9). EBV reactivation has been shown to occur in patients with various autoimmune diseases (10). Similarly, certain drugs, such as HDACIs (11), bendamustine (12) and aspirin (13), can stimulate EBV reactivation. Interestingly, infections with other pathogens can also activate latent EBV, including *Porphyromonas gingivalis* (14), Treponema pallidum (15), helminths (16), AIDS (4) and even COVID-19 (17), leading to the production of infectious viral particles that allow the virus to spread from cell to cell and from host to host. When latent EBV reacts and enters the lysogenetic phase, it again causes acute symptoms of infection (18).

Cytomegalovirus (CMV), a *β*-herpesvirus sharing transmission routes with EBV, demonstrates 60–90% global seroprevalence and typically establishes asymptomatic persistence in immunocompetent host (19, 20). *Toxoplasma gondii*, a foodborne parasite with worldwide seroprevalence exceeding 30%, poses significant risks to immunocompromised populations despite frequent subclinical courses in healthy individuals (21–23). Herpes simplex virus (HSV), comprising two subtypes with global distribution, characteristically establishes latent infections with reactivation potential (24, 25). These pathogens employ diverse immune evasion strategies – including CMV-mediated T-cell suppression, *Toxoplasma gondii*’s parasitophorous vacuole formation, and HSV blockade of antigen presentation – that collectively contribute to their persistence and potential for co-infection (26–28).

It is well known that EBV, *Toxoplasma gondii,* HSV, and CMV share similar latent and reactivating biphasic life cycles. Although some studies have examined the serological associations between EBV and other herpesviruses, the comprehensive association between EBV reactivation and antibodies against *Toxoplasma gondii*, CMV, and HSV warrants further exploration (29, 30). In this context, the aims of this study were (1) to assess the epidemiology of EBV, CMV, HSV, and *Toxoplasma gondii* and the relationship between EBV infection and the positivity rates of antibodies against these three pathogens and (2) to associate the EBV antibody status with antibodies against the three pathogenic microorganisms. These investigations will provide researchers and clinicians with more baseline information, which will aid in the diagnosis and prevention of EBV reactivation and the occurrence and development of related microbial infections.

## 2 Subjects and methods

### 2.1 Ethics approval and consent to participate

This retrospective study was approved by the Institutional Ethics Committee of the First Affiliated Hospital of CQMU (Approval No.: K2023-217) and was conducted in full compliance with the ethical principles set forth in the Declaration of Helsinki. Given the retrospective nature of the study and the anonymization of patient data, the requirement for informed consent was waived. Patient identifiers were securely and permanently anonymized before the analysis of the test results to ensure the confidentiality and privacy of the study participants.

### 2.2 Patients and samples

**Figure 1:**
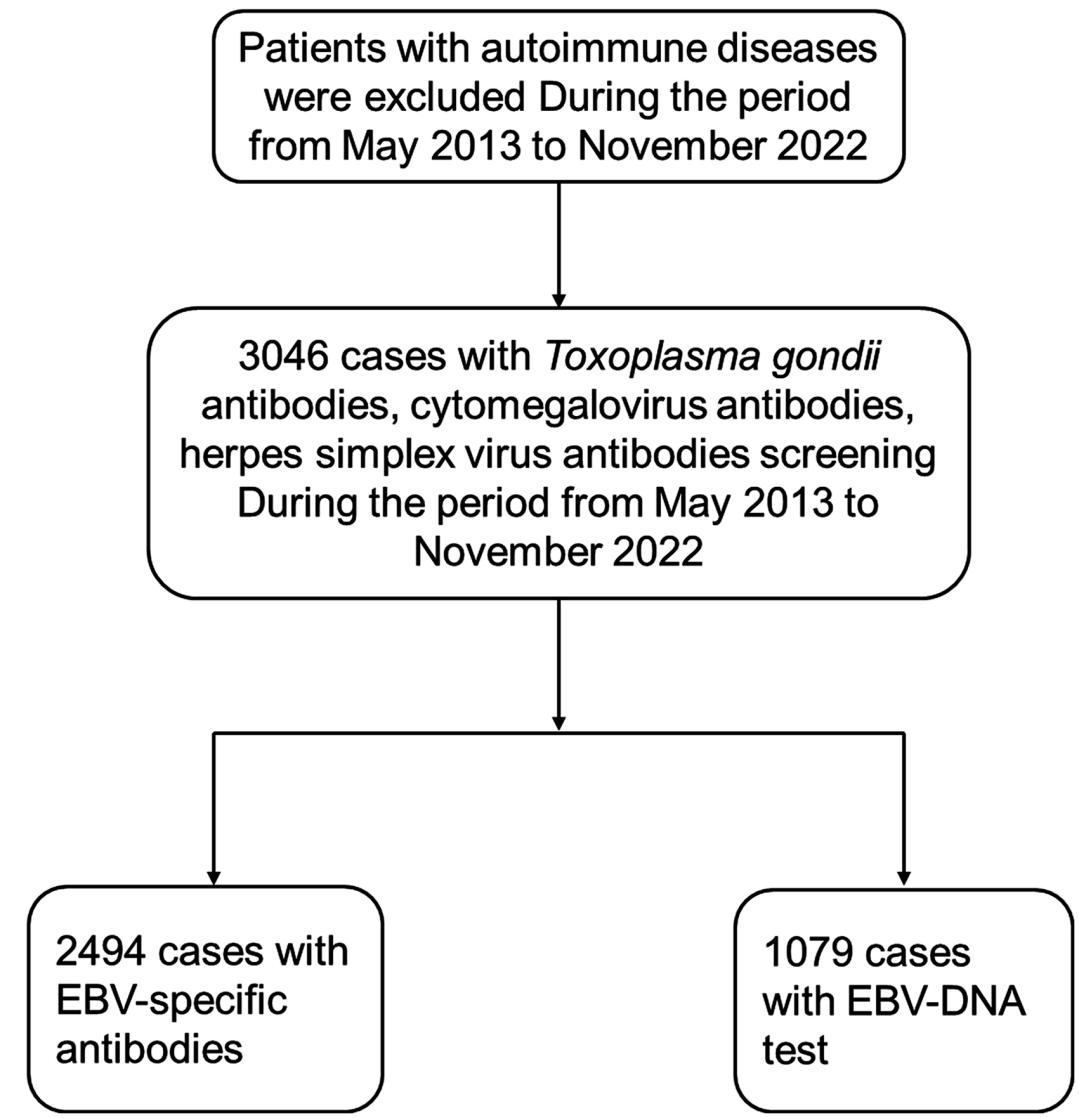
Description of the flowchart illustrating the process of selecting the study population. EBV, Epstein–Barr virus.

This retrospective study analyzed 3,046 hospitalized patients (1,524 males and 1,522 females) recruited from the First Affiliated Hospital of Chongqing Medical University, a large general hospital in Chongqing, China. The participants underwent serological testing for *Toxoplasma gondii*, cytomegalovirus (CMV), herpes simplex virus (HSV), and Epstein–Barr virus (EBV) antibodies, as well as EBV-DNA profiling (Figure 1). It is important to note that this testing was not part of a general admission screening protocol, nor was it restricted to patients with suspected infectious diseases; instead, tests were performed based on a combination of clinical indications at the physician’s discretion, such as fever of unknown origin, suspected mononucleosis, or preoperative screening. Not all enrolled patients underwent testing for every antibody or EBV-DNA due to the retrospective nature of this study.

### 2.3 Laboratory testing

Antibodies against EBV nuclear antigen IgG (EBNA-1-IgG), EBV capsid antigen IgM (EBV-VCA-IgM), EBV early antigen IgG (EBV-EA-IgG), EBV early antigen IgA (EBV-EA-IgA), EBV capsid antigen IgA (EBV-VCA-IgA), *Toxoplasma gondii* antibodies, cytomegalovirus antibodies, and herpes simplex virus antibodies were measured using an automated enzyme-linked immunosorbent assay system (Sysmex Europe, HISCL-5000). Due to detection limitations, this study could not distinguish between HSV subtypes. Quantitative real-time polymerase chain reaction (RT–PCR) was performed to detect EBV-DNA (Antpedia, AGS4800).

#### 2.3.1 EBV DNA quantification by real-time qPCR

Whole blood samples from patients were centrifuged to separate plasma and cellular fractions. Peripheral blood mononuclear cells (PBMCs) were isolated from the cellular fraction via Ficoll–Hypaque density gradient centrifugation. DNA was extracted from PBMCs (10^6^ cells) or plasma (200 μL) using the QIAamp DNA Mini Kit (Qiagen, Germany). EBV DNA was quantified with the EBV DNA Real Time PCR Kit (Liferiver Bio-Tech Ltd., Shanghai, China) on the Antpedia AGS4800 system.

The PCR amplification protocol included: 37 °C for 2 min, 94 °C for 2 min, followed by 40 cycles of 93 °C for 15 s and 60 °C for 60 s. The limit of detection was 500 copies/mL plasma or 500 copies per 10^6^ PBMCs.

Definitions of EBV infection status:

Testing for anti-EBV-EBNA-1-IgG, anti-EBV-VCA-IgM, and anti-EBV-EA-IgG in all samples covered different stages of EBV infection. The definitions of EBV infection were as follows:

No infection: Negative for anti-EBV- EBNA-1-IgG, anti-EBV-VCA-IgM, and anti-EBV-EA-IgG.

Primary infection: Positive for anti-EBV-EA-IgG and/or anti-EBV-VCA-IgM, negative for anti-EBV-NA-IgG.

Past infection: Double negative for anti-EBV- EA-IgG and anti-EBV-VCA-IgM and positive for anti-EBV-VCA-IgG and/or anti-EBV-NA-IgG.

Reactivated infection: Positive for anti-EBV-EA-IgG and/or anti-EBV-VCA-IgM and anti-EBV-NA-IgG.

### 2.4 Statistical analysis

Categorical variable data are expressed as *n* (%), and the *χ*^2^ test was used for comparisons. All statistical analyses were performed using IBM SPSS Statistics software V27.0. A two-tailed *p* value of less than 0.05 was considered statistically significant.

## 3 Results

### 3.1 EBV antibody spectrum and EBV-DNA status in different gender–age groups

As shown in Table 1, a total of 3,046 patients underwent testing for EBV-related antibody profiles or EBV-DNA. The actual number of patients for each testing parameter in each age group can be calculated by dividing the number of positive patients by the percentage. Among the patients tested for EBV antibody profiles, the overall seropositivity rates for anti-EBV-EBNA-1-IgG, anti-EBV-VCA-IgM, anti-EBV-EA-IgG, anti-EBV-EA-IgA, anti-EBV-VCA-IgA, and EBV-DNA were 90.21, 6.62, 12.43, 11.99, 24.50, and 13.90%, respectively. The seropositivity rates for males were 90.05, 7.14, 11.24, 12.97, 23.08, and 14.10%, respectively. The seropositivity rates for females were 90.38, 6.09, 13.62, 11.01, 25.94, and 13.70%, respectively. Among hospitalized patients tested for EBV-DNA, the age group most minimally affected by EBV was aged 21–40 years, with a positivity rate of 9.97%. The positivity rate gradually increased with age. The highest positivity rate was determined for adults over 60 years of age, with a positivity rate of 22.41%.

**Table 1:** Age and sex distribution of patients tested for EBV antibody profile and EBV-DNA.

	anti-EBV-EBNA-1-IgG *n* (%)	anti-EBV-VCA-IgM *n* (%)	anti-EBV-EA-IgG *n* (%)	anti-EBV-EA-IgA *n* (%)	anti-EBV-VCA-IgA *n* (%)	EBV-DNA *n* (%)
0–20 (years)	198 (75.29)	39 (14.83)	30 (11.41)	47 (18.22)	67 (25.97)	12 (11.76)
21–40 (years)	799 (90.69)	75 (8.51)	91 (10.33)	103 (11.92)	186 (21.53)	36 (9.97)
41–60 (years)	851 (93.83)	33 (3.64)	103 (11.36)	83 (9.28)	205 (22.93)	50 (13.02)
>60 (years)	402 (90.74)	18 (4.06)	86 (19.41)	61 (13.99)	143 (32.80)	52 (22.41)
Male	1122 (90.05)	89 (7.14)	140 (11.24)	159 (12.97)	283 (23.08)	76 (14.10)
Female	1128 (90.38)	76 (6.09)	170 (13.62)	135 (11.01)	318 (25.94)	74 (13.70)
Total	2250 (90.21)	165 (6.62)	310 (12.43)	294 (11.99)	601 (24.50)	150 (13.90)

anti-EBNA-1-IgG, anti-Epstein–Barr nuclear antigen 1 immunoglobulin G; anti-EBV-EA-IgA, anti-Epstein–Barr virus early antigen immunoglobulin A; anti-EBV-EA-IgG, anti-Epstein–Barr virus early antigen immunoglobulin G; anti-EBV-VCA-IgA, anti-Epstein–Barr virus viral capsid antigen immunoglobulin A; anti-EBV-VCA-IgM, anti-Epstein–Barr virus viral capsid antigen immunoglobulin M; EBV-DNA, Epstein–Barr virus deoxyribonucleic acid. Percentages are calculated based on the number of patients tested for each specific assay, as not all patients completed all tests.

### 3.2 Cytomegalovirus antibodies, herpes simplex virus antibodies and *Toxoplasma gondii* antibodies in different sex–age groups

Further observation of the seropositivity rates of *Toxoplasma gondii*, cytomegalovirus, and herpes simplex virus antibodies in 3,046 patients is shown in Table 2. We conducted tests for *Toxoplasma gondii*, cytomegalovirus, and herpes simplex virus antibodies in 3,046 patients. The overall seropositivity rates for anti-*Toxoplasma gondii* IgG, anti-CMV-IgG, and anti-HSV-IgG antibodies were 16.84, 97.50, and 91.20%, respectively. The rate of anti-*Toxoplasma gondii* IgG antibody seropositivity gradually increased with age. In patients over 40 years of age, the rate of *Toxoplasma gondii* seropositivity was significantly higher than that in those aged 0–40 years. The seropositivity rates for anti-CMV-IgG antibodies and anti-HSV-IgG antibodies also increased with age, with the lowest rates in patients aged 0–20 years of 93.19 and 73.37%, respectively. The highest seropositivity rates in patients over 60 years of age were 99.24 and 99.05%, respectively.

**Table 2:** Age and sex distribution of patients tested for anti-*T. gondii* IgM/IgG, anti-CMV IgM/IgG, and anti-HSV IgM/IgG.

	anti-*T. Gondii* IgM *n* (%)	anti-CMV IgM *n* (%)	anti-HSV IgM *n* (%)	anti-*T. Gondii* IgG *n* (%)	anti-CMV IgG *n* (%)	anti-HSV IgG *n* (%)
0–20 (years)	26 (8.05)	110 (34.06)	116 (35.91)	41 (12.69)	301 (93.19)	237 (73.37)
21–40 (years)	95 (8.77)	363 (33.52)	475 (43.86)	137 (12.65)	1046 (96.58)	939 (86.70)
41–60 (years)	66 (5.94)	331 (29.79)	327 (29.43)	227 (20.43)	1098 (98.83)	1078 (97.03)
>60 (years)	20 (3.78)	165 (31.19)	88 (16.64)	108 (20.42)	525 (99.24)	524 (99.05)
Male	121 (7.94)	432 (28.35)	440 (28.87)	278 (18.24)	1484 (97.38)	1387 (91.01)
Female	86 (5.65)	537 (35.28)	566 (37.19)	235 (15.44)	1486 (97.63)	1391 (91.39)
Total	207 (6.80)	969 (31.81)	1006 (33.03)	513 (16.84)	2970 (97.50)	2778 (91.20)

*T. Gondii*, *Toxoplasma gondii*; CMV, Cytomegalovirus; HSV, Herpes simplex virus.

### 3.3 Relationships between different EBV antibody statuses and cytomegalovirus IgG, herpes simplex virus IgG and *Toxoplasma gondii* IgG antibodies

As shown in Table 3, the positivity rates of the EBV antibody profiles differed among patients with different viral antibody statuses. In patients who were positive for anti-CMV-IgG, the positivity rate for anti-EBV-EBNA-1-IgG (90.81%) was higher than that for anti-CMV-IgG-negative patients (61.81%) (a: *p* < 0.001). In cytomegalovirus IgG-positive patients, the positivity rates for anti-EBV-VCA-IgM (6.22%), anti-EBV-EA-IgG (12.24%), and anti-EBV-EA-IgA (11.72%) were lower than those for cytomegalovirus IgG-negative patients, which were anti-EBV-VCA-IgM (25.45%), anti-EBV-EA-IgG (23.64%), and anti-EBV-EA-IgA (25.45%) (b: *p* < 0.001, c: *p* = 0.011; d: *p* < 0.001). Among patients with anti-HSV-IgG-positive disease, the positivity rate for EBNA-1-IgG (91.46%) was significantly greater than that in anti-HSV-IgG-negative patients (77.63%) (e: <0.001). In anti- HSV-IgG-positive patients, the positivity rate for EBV-VCA-IgM (5.75%) was significantly lower than that in anti-HSV-IgG-negative patients (15.35%) (f: *p* < 0.001). In anti-*Toxoplasma gondii*-IgG-positive patients, all the EBV-related antibody seropositivity rates were higher than those in anti-*Toxoplasma gondii*-negative patients, with the rates of anti-EBV-EA-IgG (15.73%) and anti-EBV-VCA-IgA (31.62%) being higher than those of *Toxoplasma gondii*-negative patients anti-EBV-EA-IgG (11.84%) and EBV-VCA-IgA (23.14%) (g: *p* = 0.032; h: *p* < 0.001).

**Table 3:** Relationship between different EBV antibody status and different virus antibody positive rate.

EBV antibody	Anti-CMV IgG			Anti-HSV IgG			Anti-*T. Gondii* IgG		
	Negative *n* (%)	Positive *n* (%)	*p*-value	Negative *n* (%)	Positive *n* (%)	*p*-value	Negative *n* (%)	Positive *n* (%)	*p*-value
Anti-EBV-EBNA-1 IgG	34 (61.81)	2,204 (90.81)	<0.001^a^	177 (77.63)	2067 (91.46)	<0.001^e^	1882 (89.87)	362 (91.88)	0.222
Anti-EBV-VCA-IgM	14 (25.45)	151 (6.22)	<0.001^b^	35 (15.35)	130 (5.75)	<0.001^f^	134 (6.40)	31 (7.87)	0.282
Anti-EBV-EA-IgG	13 (23.64)	297 (12.24)	0.011^c^	28 (12.28)	282 (12.47)	0.932	248 (11.84)	62 (15.73)	0.032^g^
Anti-EBV-EA-IgA	14 (25.45)	280 (11.72)	<0.001^d^	32 (14.22)	262 (11.79)	0.286	237 (11.52)	57 (14.65)	0.082
Anti-EBV-VCA-IgA	16 (30.19)	583 (24.41)	0.334	53 (23.55)	546 (24.58)	0.733	476 (23.14)	123 (31.62)	<0.001^h^
EBV-DNA	3 (17.65)	147 (13.84)	0.653	10 (14.28)	140 (13.86)	0.924	121 (13.96)	29 (13.68)	0.917

Statistical significance was defined as a *p* value <0.05.

*T. Gondii*, *Toxoplasma gondii*; HSV, Herpes simplex virus; CMV, Cytomegalovirus.

Anti-EBNA-1-IgG, Anti-Epstein–Barr nuclear antigen 1 immunoglobulin G; Anti-EBV-EA-IgA, Anti-Epstein–Barr virus early antigen immunoglobulin A; Anti-EBV-EA-IgG, Anti-Epstein–Barr virus early antigen immunoglobulin G; Anti-EBV-VCA-IgA, Anti-Epstein–Barr virus viral capsid antigen immunoglobulin A; Anti-EBV-VCA-IgM, Anti-Epstein–Barr virus viral capsid antigen immunoglobulin M; EBV-DNA, Epstein–Barr virus deoxyribonucleic acid.

a The serum positivity rate of anti-EBV-EBNA-1 IgG was significantly higher in anti-CMV IgG-positive patients compared to anti-CMV IgG-negative patients.

b The serum positivity rate of anti-EBV-VCA IgM was significantly lower in anti-CMV IgG-positive versus anti-CMV IgG-negative patients.

c Anti-EBV-EA IgG seropositivity was markedly reduced in anti-CMV IgG-positive compared with anti-CMV IgG-negative individuals.

d Patients positive for anti-CMV IgG demonstrated significantly lower anti-EBV-EA IgA seroprevalence than anti-CMV IgG-negative counterparts.

e Anti-EBNA-1 IgG seropositivity rates were elevated in anti-HSV IgG-positive versus anti-HSV IgG-negative subjects.

f A significant decrease in anti-EBV-VCA IgM positivity was observed in anti-HSV IgG-positive compared to anti-HSV IgG-negative groups.

g Anti-EBV-EA IgG positivity rates were higher in anti-*Toxoplasma gondii* IgG-positive patients than in anti-*Toxoplasma gondii* IgG-negative controls.

h Anti-EBV-VCA-IgA seroprevalence showed significant elevation in anti-*Toxoplasma gondii* IgG-positive versus anti-*Toxoplasma gondii* IgG-negative cohorts. Percentages are calculated based on the number of patients tested for each specific assay, as not all patients completed all tests.

### 3.4 Cytomegalovirus IgM, herpes simplex virus IgM and *toxoplasma gondii* IgM antibodies in different EBV infection states

Among the 3,046 patients who underwent EBV antibody profile testing, four distinct EBV infection statuses were classified on the basis of antibody combination patterns: 167 patients were classified as uninfected, 77 patients had primary infections, 1,908 patients had past infections, and 342 patients experienced reactivation infections. As shown in Table 4, the positivity rate of anti-CMV-IgM antibodies in patients with EBV reactivation (60.53%) was significantly greater than that in uninfected patients (25.75%), primary infection patients (45.45%), and past infection patients (29.04%) (a: *p* < 0.001; d: *p* = 0.016; c: *p* < 0.001). The positivity rate of anti-CMV-IgM antibodies in primary EBV infection patients (45.45%) was higher than that in uninfected patients (25.75%) (b: *p* < 0.001). The positivity rate of anti-HSV-IgM antibodies in primary infections was higher than that in uninfected patients (*p* = 0.059).

**Table 4:** Relationship between different EBV infection status and different virus IgM antibody positive rate.

Antibody spectrum combination patterns (Anti-EBNA-IgG, Anti-EBV-VCA-IgM, Anti-EBV-EA-IgG)	Infection status	Anti-CMV-IgM-positive *n* (%)	*p*-value	Anti-HSV-IgM-positive *n* (%)	*p*-value	Anti-*T. Gondii* -IgM-positive *n* (%)	*p*-value
−/−/−	No infection	43 (25.75)	<0.001^a^	53 (31.74)	0.131	12 (7.19)	0.946
−/+/+, −/+/−, −/−/+	Primary infection	35 (45.45)	<0.001^b^	30 (38.96)	0.059	6 (7.80)	0.866
+/−/−	Past infection	554 (29.04)	<0.001^c^	612 (32.08)	0.612	156 (8.18)	0.658
+/+/+, +/+/−, +/−/+	Reactivation	207 (60.53)	0.016^d^	132 (38.60)	0.853	25 (7.31)	0.614

Statistical significance was defined as a *p* value <0.05.

EBV, Epstein–Barr virus; *T. Gondii*, *Toxoplasma gondii*; HSV, Herpes simplex virus; CMV, Cytomegalovirus.

Anti-EBNA-1-IgG, Anti-Epstein–Barr nuclear antigen 1 immunoglobulin G; Anti-EBV-VCA-IgM, Anti-Epstein–Barr virus viral capsid antigen immunoglobulin M; Anti-EBV-EA-IgG, Anti-Epstein–Barr virus early antigen immunoglobulin G.

a The Anti-CMV IgM seropositivity rate in EBV reactivation patients (60.53%) was significantly higher than in EBV-uninfected controls (25.75%) (*p* < 0.001).

b Anti-CMV IgM positivity was elevated in primary EBV infection cases (45.45%) compared to EBV-uninfected controls (25.75%) (*p* < 0.001).

c EBV reactivation patients (60.53%) exhibited markedly higher anti-CMV IgM seropositivity than past EBV infection cases (29.04%) (*p* < 0.001).

d A significant difference in anti-CMV IgM positivity was observed between EBV reactivation patients (60.53%) and the primary EBV infection cohort (45.45%) (*p* = 0.016). Percentages are calculated based on the number of patients tested for each specific assay, as not all patients completed all tests.

## 4 Discussion

When the human body is initially infected with a virus, the primary infection is usually cleared, resulting in subclinical symptoms (31). However, viruses from families such as Herpesviridae, Polyomaviridae, Adenoviridae, and Parvoviridae can become latent, leading to stable maintenance of the latency of the virus in healthy individuals (10). EBV infection is extremely common worldwide, and its infection status varies by age, ethnicity, country, and region. Since its initial discovery, its threat to human health has become increasingly known (3). The National Institutes of Health (NIH) has identified EBV control as critical for reducing the global cancer burden (32).

In this study, the prevalence of EBV VCA-IgM in the population aged 0–20 years was 14.83%, a rate that shares similar epidemiological characteristics with the 11.23% seroprevalence reported in a similar study conducted in Beijing (33). Additionally, the higher positive rate among hospitalized patients in our study may be associated with their pathological conditions. Age is an important risk factor for EBV infection, and our research revealed that the positive rate of EBV-DNA was lowest in the 21–40 age group (9.97%), increasing after the age of 40 years. These findings indicated that acute or reactivated EBV infections occurred primarily in children, adolescents, and elderly individuals. Current studies suggest that children are the main population for acute EBV infection or reactivation, as their immune systems are immature (34, 35). As the immune system matures with age, the incidence of EBV reactivation decreases (36). In contrast, elderly individuals experience a gradual decline in immune function, resulting in an increased incidence of EBV reactivation.

In this study, we detected *Toxoplasma* antibodies in the serum of 3,046 participants using an automated enzyme-linked immunosorbent assay, revealing an overall seroprevalence of *Toxoplasma* IgG antibodies of 16.84%, which was higher than that revealed in a national survey conducted from 2000 to 2017, which reported a prevalence of 8.20% (37). However, the seroprevalence reported in this study was lower than the overall prevalence reported in Europe of 32.1% (38). This discrepancy might be related to economic development and improvements in quality of life as the awareness of health and hygiene increases. This study also revealed that seroprevalence significantly increases with age, which was consistent with findings from research conducted in Egypt (39). These results suggest that most *Toxoplasma gondii* infections are acquired through postnatal transmission, with cumulative exposure over a lifetime increasing the likelihood of infection. This study revealed that patients who were positive for *Toxoplasma* IgG antibodies had higher positive rates of EBV-EA-IgG (15.73%) and EBV-VCA-IgA (31.62%) compared with the control group, which was negative for *Toxoplasma* IgG antibodies, with rates of 11.84% for EBV-EA-IgG and 23.14% for EBV-VCA-IgA. This finding was distinct from the results related to cytomegalovirus and herpes simplex virus, potentially owing to changes in the immune response of patients following prior *Toxoplasma* infections.

The activation status of EBV was subsequently classified on the basis of serum antibody profiles. The presence of IgM antibodies against herpes simplex virus and *Toxoplasma gondii* was not significantly correlated with the incidence of EBV reactivation. This discrepancy may stem from the pathogens’ distinct neurotropic properties, particularly exemplified by HSV-1. The neurotropism of HSV is manifested through its unique capacity for retrograde axonal transport along sensory neurons to dorsal root ganglia, enabling lifelong latent infection establishment. This process is initiated by specific interactions between viral glycoprotein gD and neuronal nectin-1 receptors, mediated through a microtubule-dependent intracellular transport system. In contrast, while *Toxoplasma gondii* lacks HSV’s strict axonal retrograde transport machinery, it achieves central nervous system (CNS) invasion via multi-faceted strategies, exhibiting unique and sophisticated pathogen-host interaction networks during neural chronic infection processes (28). However, we had found that a significant relationship was identified between positive IgM antibodies for cytomegalovirus (CMV) and EBV reactivation, which has the potential to aid in the clinical prevention of EBV-related diseases. There is a considerable likelihood that EBV and CMV share common mechanisms of coinfection, as both belong to the herpesvirus family (40). Coinfection with EBV and CMV in transplant recipients may lead to the following consequences: persistent EBV replication can promote abnormal expression of immunoglobulin heavy chains in B cells, while active CMV infection may exacerbate clonal proliferation of EBV-infected B cells through immunosuppression or alterations in the immune microenvironment (41). The incidence of graft dysfunction, rejection, and acute rejection in coinfected patients is significantly higher than in non-infected groups (42). Numerous researchers have extensively investigated the associations between CMV and various disease conditions (43). CMV seropositivity has been linked to cardiovascular disease (CVD) (44), rheumatoid arthritis (45), diabetes (46) and overall mortality (47). In summary, we found that the antibody profile of EBV reactivation in hospitalized patients is related to anti-CMV-IgM, which is consistent with Research by others: CMV is a particularly active inducer of EBV reactivation (48). To date, no pathogen that exerts an effect on the immune system similar to that of CMV has been described (49). For example, EBV does not induce overall changes in immune phenotypes in the same manner as CMV (19). One possible explanation for this phenomenon is that CMV infection has profound effects on many components of the immune response, which in turn influences the response to EBV and other viruses (50). It is currently hypothesized that autoimmune responses may be activated during CMV infection (51), which could explain why the rate of EBV reactivation is higher in CMV-IgM-positive patients than in other groups.

This study has several limitations inherent in its single-center cross-sectional design, including the absence of longitudinal data, limited multidimensional assessment, and lack of mechanistic investigations. Future multicenter studies with larger sample sizes should validate the epidemiological patterns observed here and further explore the interactions between EBV reactivation and CMV/*Toxoplasma gondii* seropositivity through longitudinal monitoring and mechanistic approaches.

This hospital-based study establishes crucial baseline seroprevalence data for EBV, CMV, HSV, and *Toxoplasma gondii* co-infections in Southwest China, revealing distinct age-specific patterns and significant associations between EBV reactivation and CMV/*Toxoplasma gondii* seropositivity. These findings provide valuable epidemiological references for optimizing diagnostic strategies and guiding future research on herpesvirus and parasite co-infections in clinical populations.

## 5 Conclusion

EBV antibody profiles positivity rates were higher in patients with *Toxoplasma gondii* IgG positivity than in those with *Toxoplasma gondii* IgG negativity. The CMV IgM antibody positivity rate was significantly higher in EBV reactivation group than in other groups.

## Data Availability

The original contributions presented in the study are included in the article/supplementary material, further inquiries can be directed to the corresponding author.
